# A qualitative study on teachers’ perceptions of their learners’ mental health problems in a disadvantaged community in South Africa

**DOI:** 10.4102/curationis.v42i1.1903

**Published:** 2019-11-27

**Authors:** Donald Skinner, Carla Sharp, Lochner Marais, Motsaathebe Serekoane, Molefi Lenka

**Affiliations:** 1 Research on Health and Society, Faculty of Health Sciences, Stellenbosch University, Cape Town, South Africa; 2 Social Aspects of Public Health, Human Sciences Research Council, Cape Town, South Africa; 3 Department of Psychology, University of Houston, Houston, United States; 4 Centre for Development Support, University of the Free State, Bloemfontein, South Africa; 5 Department of Anthropology, University of the Free State, Bloemfontein, South Africa

**Keywords:** orphans, vulnerable children, South Africa, HIV, poverty

## Abstract

**Background:**

The combination of extensive poverty, violence and HIV has potential mental health impacts on children in Southern Africa. This article is nested in a broader study to evaluate the strength and difficulties questionnaire (SDQ) among Sotho speakers, and assess the mental health status of children made orphans by AIDS.

**Objectives:**

The aim of this study was to describe the mental health problems that the teachers perceive among learners in their classrooms, to understand what the teachers saw as causing these problems and to identify potential approaches to address these problems within the school setting.

**Method:**

As part of the larger study, 10 teachers were purposively selected to write a report describing the mental health problems among learners in their class. These findings were discussed at two later meetings with a larger grouping of teachers to validate the findings and obtain additional input.

**Results:**

The teachers were concerned about the emotional state of their pupils, especially in relation to depression, anxiety, substance abuse, scholastic problems and aggression. These problems were felt to arise from the children’s lived context; factors such as poverty, death of parents and caregivers from AIDS and trauma, parental substance abuse and child abuse. The teachers expressed a desire to assist the affected learners, but complained that they did not get support from the state services.

**Conclusion:**

Many learners were evaluated by teachers as struggling with mental health issues, arising from their social context. The teachers felt that with support, schools could provide assistance to these learners.

## Introduction

Human immunodeficiency virus (HIV), poverty-heightened levels of substance abuse and community and family violence have led to the creation of a large group of disadvantaged children in sub-Saharan Africa, including South Africa (SA). While these conditions are not characteristics for all children in SA, evidence of such traumatised and deprived children is found in many of the poorer and more disadvantaged communities. The impact of orphanhood because of HIV on children in the developing world is well documented (Boyes & Cluver 2013; Cluver 2011; Cluver, Bowes & Gardner 2010; Cluver & Gardner 2007a; Cluver, Gardner & Operario 2008; Foster, Levine & Williamson 2005; Sharer et al. 2016: Wild 2001). Over the last 10 years, several cross-sectional studies in SA (Cluver & Gardner 2007b; Cluver, Gardner & Operario 2007) and other developing countries (Bhargava 2005; Doku 2016; Dorsey et al. 2015; Fang et al. 2009; Gong et al, 2009; Hapunda 2015; Kumar et al. 2014; Lata & Verma 2013; Li et al. 2009; Makame, Ani & Grantham-McGregor 2002; Nyamukapa et al. 2008; Puffer et al. 2012) have demonstrated high rates of psychopathology, especially internalising disorders in children orphaned by HIV and AIDS. Longitudinal studies confirm the persisting negative impact of orphanhood in SA (Cluver et al. 2012).

While orphaned children are felt to be particularly at risk, it is important to look at the mental health risks of children made vulnerable by all causes, including children rendered vulnerable by parents’ desertion or illness, by parental neglect because of substance abuse or for other reasons, such as poverty, poor housing, violence and other causes that make it harder for the children to reach their potential or put them at a serious disadvantage in life (Skinner et al. 2006). Many of the South African studies listed above compared, on the basis of mental health, children orphaned with those not orphaned living in the same community. While the mental health issues were generally less in the latter group, they still raised concern and would warrant a general community intervention. Given the levels of poverty and other social problems in this community, the research team in the current study considered most of the children in target field site to be vulnerable (Sharp et al. 2014).

There is much more limited research drawing on the insights of teachers who are able to observe the learners for an extended period during the school term; one paper was found published subsequent to the fieldwork for this study (Mfidi 2017). Work in Swaziland has argued for the potential importance of schools in assisting children affected by HIV, such as provision of food, protection and contributing to the children’s socialisation (Nordtveit 2010; Poulson 2006).

The study was conducted in a large township in the urban centre of Mangaung, the capital of the Free State, located in the heart of SA. The Free State is one of the areas most affected by HIV with a prevalence rate of 25.5% among adults aged 15–49 years (Human Sciences Research Council 2018). The Free State is the third largest province in SA and covers 10.6% of the surface area. It is also the third most urbanised province, with the Mangaung Local Municipality in the Motheo District being the most densely populated. Of the 752 906 people living in Mangaung, 618 408 (82%) are black people (mostly Sotho speaking), 101 170 (14%) are white people, 32 071 (4%) are people of mixed race and 1257 (0.2%) are of Indian or Asian descent. These are formal racial categories used for research and for employment legislation. In 2016, 20.8% of children in the Free State lived with neither parent and only 37.1% lived with both parents (Jamieson, Berry & Lake 2017). The aim of this study was to describe the mental health problems that the teachers perceive among learners in their classrooms, to understand what the teachers saw as causing and maintaining these mental health problems and to identify potential approaches to address these problems making use of the school setting. The study looked at the general mass of learners and was not limited to those who had been orphaned.

## Methodology

This article is nested within a larger study aimed at evaluating the psychometric properties of the strength and difficulties questionnaire (SDQ) (Goodman 1997, 2001) for use among a Sotho-speaking population in SA (Sharp et al. 2014). In this larger study, in addition to the psychometric evaluations performed by parents and the children themselves, the authors consulted with the teachers of these children to understand how they see the mental health problems of learners in their classrooms. The teachers’ evaluations were based on their observations of these children over an extended period. Teachers have regular contact with large numbers of children, and in the presence of a good teacher–learner relationship, the children will often confide in their teachers. For this reason, teachers were considered to be good informants of the kind of problems that their learners face. In a separate paper, the teachers evaluated the SDQ as an assessment instrument and they were extremely positive about their capacity to use the tool and its potential to assist them in assessing and supporting the learners in their classrooms (Skinner et al. 2014).

For the main study, children, aged 7–11, who had been orphaned as a result of HIV, were identified through organisations that provide care and support to their households. The children, their primary caregivers and their teachers were all asked to assess the emotional and behavioural problems of the children using the SDQ relevant for that age group. Teachers were approached by the study fieldworkers, and if they agreed to participate, they were given a brief training in the use of the SDQ. Each teacher was given a list of learners in their class that they were asked to observe and assess using the SDQ. The teachers were paid a small honorarium of R100 per learner. They were given up to 2 weeks to complete the assessment and the forms were then collected by the fieldworkers. While the completion of the SDQ assessments did not directly relate to the fieldwork for this study, by completing them, the teachers became more aware of the mental health issues affecting their students. The fieldworkers were drawn from these communities so had insight into the context of the learners’ life and were extensively trained in all aspects of the study.

### Participants

After having completed about two-thirds of the total assessments for the study, so having given the teachers a number of opportunities using the tool, 10 teachers who had each assessed at least five learners were approached to write an evaluation report on their experience of using the SDQ and on their assessments of the mental health concerns of their students. The inclusion criteria for the original group of teachers to complete SDQ forms on their learners were that they taught grades 3–6 for at least 3 years at schools in the community where the research was being performed, and had daily contact with pupils. The selection of the teachers to complete the evaluation report was on the basis of obtaining a spread across multiple schools in the communities where the study was conducted and having a spread across grades 3–6, and those who the fieldworkers felt had shown the greatest insight into objectives of the broader study. Each teacher was paid R500 to write a report of about five pages.

### Measures

The terms of reference that the teachers responded to first asked them to comment on the SDQ itself, which is reported in a separate paper (Skinner et al. 2014). Then, they were asked to describe, in two pages, the nature of the mental health problems that learners in their school faced, how prevalent these problems were, how seriously they affect the learners’ capacity to work and what services were available to assist these children. The teachers were specifically asked the following questions:

- We wish you to describe the nature of the mental health problems that learners in your school face, how prevalent these problems are and how seriously they affect the learners’ capacity to work.
- What are the mental health problems that learners face? For example, depression, anxiety, aggression, concentration problems, substance abuse, mourning and so on.
- Are some more prevalent than others?
- Do most children with mental health problems come from one group?
- What services currently exist to assist these learners?
- How accessible are these services and how many children use them?
- How do these problems impact on the learners’ lives, social interactions and school work?

Once all the reports had been gathered and a provisional analysis completed, two public meetings were held with teachers from all schools included in the study. These meetings were held at a central location and at times convenient for the teachers to ensure that those who wished were able to attend. At this meeting, the findings were presented and the teachers affirmed the findings, expanding particularly on the issue of providing care and support for the pupils. These meetings were seen as being important for assessing the validity of the findings, as the group was much larger, about 60 teachers across both meetings, than those who completed reports.

The authors acknowledge that there is possibly a bias in the teachers’ assessment, especially as they are not mental healthcare professionals. So, this article seeks their perspective on the issue and should not be treated as having diagnostic certainty. Their use of the SDQ would have sensitised the teachers to the mental health concerns and made it easier for them to identify specific mental health problems.

### Qualitative analysis

The reports were analysed using a contextualised interpretative analysis approach (Skinner 2014; Terreblanche et al. 2010), which drew together teachers’ comments into the key areas as organised in the Results section. The sections as presented in the results below follow the key themes that emerged, namely the key mental problems, teachers’ assessment of the causes of these problems, the impact of the mental healthcare problems for the learners and the teachers desire to respond effectively. The coding was performed manually as the sample was small and the reports were focused and short. Quotes were drawn from the reports written by the teachers. The quotes are indented in the text to differentiate them from the authors’ writing. No personal details on any of the teachers or learners are reported to protect confidentiality.

### Ethical consideration

Ethical clearance was obtained from the Stellenbosch University Health Research Ethics Committee (IRB00052239).

## Results

Teachers, by virtue of their position and time spent with their learners, identified a broad range of serious mental health concerns among their learners. On the basis of the teachers’ knowledge of the children, they were able to make important comments on both the causes of these problems and their impacts.

### Mental health problems among learners

The major mental health problems identified by teachers were depression, anxiety, aggression, drug addiction and scholastic problems particularly concentration, with some learners having more than one of these problems. While not giving any measures of prevalence, the impression was that these problems are widespread, and generally had a major impact on the learners and the classroom.

The depressed learners were described as having no motivation, being sad and crying in class, having no energy, not being able to establish friends and struggling to focus and remember the lessons taught. Anxiety in learners was described as problems of restlessness and overactivity, as well as the more traditional anxiety-focused problems of heightened fears, lack of impulse control, problems in socialising, distrust of fellow learners and teachers and having difficulty in focusing. Concentration problems, while raised separately, were often described as being a result of depression and anxiety. All of these disrupted the learners’ capacity to work effectively in the classroom, and even to interact socially with other learners. The teachers expressed concern that as the learners’ school experience was compromised, these problems would go further to inhibit the learners’ future.

Aggressive learners, usually the boys, were seen as being more troublesome than those with other perceived mental health problems. They were described as having difficulties controlling their emotions, especially anger and anxiety, and would therefore act out aggression and by fighting. As a result, they had a major effect on the learning situation, by disrupting classes, and creating fear with even the teachers being concerned that they could also become the focus of attacks:

‘The aggressive learner is always problematic even during the class discussions. He always draws the attention of the teacher as well as the other learners to his negative behaviour. And he is a bully to the other learners.’ (Female, primary school teacher)

Teachers reported that they saw multiple signs of drug addiction among their pupils, including use of alcohol, dagga and sniffing glue or petrol. They were reported as often, but not always, being secondary to the depression and anxiety. The combination of mental health problems and substance abuse made the behaviour of the learners worse. The teachers raised concern over the impact of this for the future of the learners, and at the social and home conditions that lead to such high and open use of substances.

### Aetiology of mental health problems

The teachers had very clear ideas about why the learners presented these problems. The mental health problems were broadly seen as stemming from the poor living conditions of the children, the lack of parenting and the high levels of trauma that they faced. The line of causation is not clearly defined, but the teachers saw direct connections between what they saw as raising stress and problems for the learners, and all the areas of pathology described above. The direct process varied at times, but there was a consistent shared understanding of the connection between context and mental health problems:

‘They are depressed from [*sic*] their daily experiences around them like; the death of their parents at young age, poverty with jobless parents; and other learners are raped and situations like these traumatise them and lead to depression. Other learners are depressed by the HIV status of their parents and all these also lead to poor concentration.’ (Female, primary school teacher)

The communities in which the children lived are impoverished because of lack of infrastructure and resources, for example, electricity, running water and sanitation for households. The parents of many of the children were unemployed and reliant on social grants that were generally not enough to support the family. Crime and violence were very high in these communities, which further increased the stress on the children and raised levels of anxiety. The teachers remarked particularly on the highly destructive implications of sexual and other physical abuse. Teachers reported that it was very difficult to know how prevalent this abuse was, as the learners often try to hide their experience of abuse, particularly the boys.

‘Schools are having children from various backgrounds such as broken families, child headed families, poverty backgrounds and so on. These various backgrounds impact negatively on the child’s mind. They impinge on learning and teaching.’ (Female, primary school teacher)

The impact of the loss of family members because of AIDS, other illnesses or trauma, was felt at two levels: the experiences of the losses and for many the subsequent lack of caring that the children received from the replacement caregivers who were even on occasion abusive. In the worst-case scenario, parent’s deaths led to child-headed households. Some teachers also reported learners becoming aggressive after losing a parent to HIV, as they got angry thinking about their losses. While the children themselves were HIV-positive, this contributed to them being depressed, because of the impacts of both the disease and fears for their future:

‘The death toll that is caused by AIDS is another tool which contributes to the mental health problems. The number of orphans is too high. Most of these orphans are kept in care centres. And unfortunately the people who take care of these children are so abusive to these kids. They do not give them love. These people are not there for taking care, but to get a salary at the end of the month.’ (Female, primary school teacher)

Teachers raised the impact of high levels and widespread abuse of alcohol and other substances by caregivers, leading to abuse and a lack of concern or guidance for the children either at a material or emotional level. Many parents abandoned their roles, leaving the children to care for one another. Some children also manifested foetal alcohol syndrome:

‘Sometimes you’ll find that both of the parents are alcoholics. So, no one is able to take care of these kids, to prepare anything for school. Such kids are always the victims of abuse.’ (Female, primary school teacher)

Peer pressure contributed greatly to substance abuse especially in contexts where parental influence is low or non-existent. As substance abuse increased, so did bullying and theft. Once the learners began using substances, it was harder to stop even if they wished to, as they were afraid of their peers. Some learners, drawing on parental examples, also used substances to dull the pain in their lives. What concerned the teachers was the young age at which the use started, even from when they first started school in a few cases. This made the patterns of use difficult to change which had implications for their schooling and their lives in general:

‘Because of the learners not having parental guidance they grow up not positively guided. Learners will grow in the midst of friends that will lead to a peer-pressure, at the end of the day substance abuse come [*sic*] into picture. When substance abuse becomes habitual we as teachers will see the signs of stealing and bullying come into picture.’ (Female, primary school teacher)

Concentration problems were a manifest part of a number of the diagnoses described above: depression, anxiety, substance use and heightened aggression. However, the teachers felt that this was aggravated by the social conditions including poor nutrition and health problems such as poor eye sight, hearing problems and other physiological problems. Some children are also exhausted by the responsibility of caring for their sick parents, their siblings or being unable to sleep because of poor housing.

### Impact of mental health problems on children

The teachers mainly described the impact of the mental health problems on the learners’ education with children struggling more and more to be able to maintain their progress. Children, who are depressed or anxious, find concentration difficult, start to fall behind and become frustrated and, in turn, get angry and aggressive. Many of the children manifesting such problems do not or are unable to pay attention in the class, do not take part in class activities, skip many days of school and so fail grades. Some learners have been advanced to higher grades because of age without having grasped basic skills including reading and writing, which further increases their frustration. Teachers were also concerned about the social impact of the mental health problems as affected learners became more isolated. Teachers stressed that in assessing the impact on the children, mental health problems must be seen in conjunction with the societal and contextual problems, especially poverty. Some specific implications identified included dropping out of school early, long-standing substance abuse, heightened aggression – as a form of coping – ingrained into their personality, movement to the streets to live or into gangs and pregnancy for girls.

### Services available

Teachers wanted to assist the learners, and provided support to them in the classroom. They also felt the need to be able to refer learners for further intervention, but these resources are extremely limited. The major points for referrals are programmes run at schools or by the Department of Education, but are very limited and focused on educational assistance rather than mental health. Professional services are available, but extremely difficult to obtain without the resources to enter private care. The referral process to any services was extremely slow with the demand being much higher than what was possible to provide given existing resources. This was reinforced in the meetings with teachers, saying that they waited years for a child to be seen, and then, sometimes, the referral was refused as the child was then too old for that service.

Some schools have the School Based Support Team (SBST) where teachers and parents work together to assist children with academic problems, who have been referred by the teachers. Being in the schools, it is easily accessible and has assisted many learners. Again, the focus is on learning problems and these lacked professional assistance so could not be easily used for mental health problems. Teachers reflected a need for increased professional and counselling assistance, and for social workers and nurses to assist in the provision of this counselling and social service support for the learners and their families:

‘The people who usually come into contact with the learners are SBST, they are general teachers who sometimes lack the skills to help emotional learners and also have to concentrate on their teaching profession.’ (Female, primary school teacher)

### Validity

The R500 payment for the report could have induced teachers to report greater levels of problems so as to meet a perceived need of the researchers. The responses did in many respects match the question asked of them in their initial task, so the question could have been seen as leading. The meetings held with the teachers, many more than completed reports, did contribute to the validity of the findings. An initial report on the findings was presented to the teachers who attended and they reinforced our interpretation, providing additional examples. The findings also tied in with the results of the main study that indicated high levels of depression, anxiety, substance abuse and trauma-related problems.

## Discussion

Teachers were shown to be aware of the mental health problems faced by the learners in their classroom, and were, in turn, concerned for them. This supports the results of another study conducted in the Eastern Cape again using teachers to identify mental health problems (Mfidi 2017). That study identified inappropriate handling of emotions, risk taking and disruptive behaviour as the core concerns (Mfidi 2017). These are similar to the behaviours described by teachers in this study. The use of the SDQ did greatly facilitate their assessment capacity (Skinner et al. 2014) and could be used more extensively. Teachers were extremely concerned about the nature and prevalence of mental health problems among their learners.

Even without knowledge of family history and generic contributors, teachers identified a broad range of factors in the community, including substance abuse, violence and generalised poverty. They felt these factors contributed to the problems the learners experienced; which resonates with other work in the area (Bruijnene et al. 2019; Flisher et al. 2012; Jacob & Coetzee 2018; Mfidi 2017). The teachers did focus on contextual causes for the problems that the learners faced, based on the changes noted in the learners occurring around the same time as traumatic events or other major changes in the learners’ lives. At the meetings we held with them at the end of the study, the teachers presented multiple-case examples of learners who they had seen deteriorate over even a single year or less as the stressors in the learners life got too much to bear. They were concerned that these impacts would be permanent and impact back into their communities. Teachers are a potentially effective group to provide assistance to learners (Nordtveit 2010).

The teachers reported feeling isolated and were frustrated at the lack of services for learners with mental health problems, and by the apparent disinterest of the state departments in improving services to assist the affected learners. Efforts do need to be made to assist these learners by increasing the number and range of facilities to refer them to, and by supporting teachers in their roles (Draper et al. 2009). There are clear gaps in the services, where policy has not been adequately implemented. Mental healthcare policy states that links should be developed with educational intuitions (Department of Health 2013) and that cooperation should provide services for learners. However, the provision of mental health services is generally a national concern (Burns 2011; Dhai & Mohamed 2018).

Recommendations of this study are varied as learners face multiple challenges in SA. There is a focus on HIV and the deaths of parents through this disease, but the teachers highlight that mental health problems and extreme life stressors leading to mental health problems can be found considerably more broadly. The mental health services at professional level for children in this community are inadequate, inaccessible and in places non-existent. Teachers offered some services, but did not feel adequately qualified or have the resources to assist. This is an area where nurses could provide important and valuable assistance, especially school health nurses (Mfidi 2017). It is also important for school health nurses to note the particular role of teachers in being able to observe learners in their classrooms over time, and so be aware of changes or mental health problems that emerge. As such they could be a valuable ally to the nurses in both identifying problems when they occur and in providing ongoing support to the learners in the classrooms. Working across professions in teams could facilitate the work of both the teachers and nurses in providing mental health services.

### Limitations

A number of limitations were noted in this study. The sample of teachers selected was small and represented those who were felt to be more committed to their professional roles and the children under their care. The teachers were not trained as mental health workers or in the assessment of mental health issues. However, as argued above, their observation of their pupils over an extended period of time gives them unique insights. This study is limited to their perceptions, which are not the same as a full diagnosis by a professional mental health worker. However, the teachers’ comments around the descriptions of mood and behaviour and their comments on the potential aetiology of the problems provided some confidence that they were giving informed insights.

## Conclusion

There are multiple mental health problems among school learners, accentuated by the disadvantaged nature of their communities, prevalence of HIV and other key diseases and the easy access to substances. Teachers can play an important role in identifying problems, providing some affirming containment and where necessary referring the pupils to other professional services. School health nurses could be a valuable ally in mental healthcare service provision to learners and this relationship should be enhanced. However, at present, there is a severe shortage of services available to these pupils, which is raising frustration among the teachers who feel unsupported in their role.
